# Does Large Needle Aspiration Biopsy Add Pain to the Thyroid Nodule Evaluation?

**DOI:** 10.1371/journal.pone.0058016

**Published:** 2013-03-11

**Authors:** Angelo Carpi, Giuseppe Rossi, Andrea Nicolini, Giorgio Iervasi, Matteo Russo, Jeffrey Mechanick

**Affiliations:** 1 Department of Clinical and Experimental Medicine, University of Pisa, Pisa, Italy; 2 National Research Council (CNR), Unit of epidemiology and Biostatistics, Institute of Clinical Physiology and G. Monasterio Foundation, Pisa, Italy; 3 National Research Council (CNR), Institute of Clinical Physiology and G. Monasterio Foundation, Pisa, Italy; 4 Sapienza University of Rome, Department of Experimental Medicine, Rome, Italy; IRCCS San Raffaele Pisana, Department of Cellular and Molecular Pathology, Rome, Italy; 5 Metabolic Support, Division of Endocrinology, Diabetes and Bone Disease, Mount Sinai School of Medicine, New York, New York, United States of America; The Chinese University of Hong Kong, Hong Kong

## Abstract

Thyroid large needle aspiration biopsy is disregarded because it is thought to be associated with pain. This is in contrast with our 32 years long experience. We surveyed reports of pain in patients examined with fine needle aspiration biopsy (78, 87.2% women, mean age 59 years) or FNAB+large needle aspiration biopsy (48, 87.5% women, mean age 60 years). Each patient was questioned regarding a) no unpleasant sensation (score “0”); b) unpleasant sensation (“1”); c) mild pain (no analgesic used; “2”); or d) pain (analgesic used; “3”). The mean size of the needle used was for FNAB 22.3±0.7 or 20.8±1 gauge in the fine needle aspiration or fine needle aspiration plus large needle aspiration biopsy group, respectively (p<.0001). The number of percutaneous punctures was higher in the fine needle aspiration plus large needle aspiration biopsy group. However, the pain score in the fine needle aspiration biopsy or fine needle aspiration biopsy plus large needle aspiration biopsy group was not significantly different. Large needle aspiration biopsy after fine needle aspiration biopsy does not add any discomfort or pain and therefore in light of the demonstrable benefits, should be included in clinical algorithms for the evaluation of thyroid nodules.

## Introduction

Fine needle aspiration biopsy (FNAB) is the principal test in the preoperative selection of thyroid nodules [1]. However, the performance of FNAB is compromised when there is inadequate cellularity or indeterminate cytological findings, arguing for complementary diagnostic procedures in these circumstances [1], [2]. Many tests have been suggested, including large needle biopsy (LNB) [2]. However, the 2006 clinical practice guidelines from the American Association of Clinical Endocrinologists [3] have not recommended the use of LNB since this procedure is thought to be cumbersome, associated with pain and bleeding, and not associated with a higher diagnostic accuracy than FNAB.

More recent clinical practice guidelines [4] regarding thyroid nodules confirmed that LNB is not recommended.

As it turns out, clarifying the specific LNB procedure used may ultimately enhance the clinical algorithm for the evaluation of thyroid nodules. Traditionally, LNB provides tissue cylinders or fragments for conventional histological evaluation [5]. These techniques can be grouped into two methods defined as large needle cutting biopsy (LNCB; or coarse needle biopsy) or large needle aspiration biopsy (LNAB) [5]. LNCB techniques employ relatively large needles: 14 gauge 2^3/8^ inch, 3^1/8^ inch Silverman, or 14 gauge 3 inch disposible Tru-Cut disposable [5]. The operator maintains sterility as a skin nick is performed to permit the insertion of the needle [6]. The LNCB technique has been nearly abandoned because it requires a sterile environment [5].

The LNAB technique was first reported in 1930 [7] and is very similar to that used for FNAB. No incision of skin is performed. Needles of different sizes can be used (usually from 18 to 20 gauge) according to the size and consistency of the nodule. In 1980, we started using LNAB with needle sizes usually ranging from 18 gauge for large nodules (>35 mm) to 22 gauge for small nodules (<10 mm) [8], [9].

This study evaluates whether LNAB adds pain to the thyroid nodule evaluation.

## Materials and Methods

### Patients

Seventy-eight patients (87.2% women, mean age 59 years) had a palpable nodules (63% single, 37% multuple nodules) examined only by FNAB. Another group of forty-eight patients (87.5% women, mean age 60 years) had nodules (58% single, 42% multiple) examined by FNAB cytology+LNAB histology. At a subsequent visit, each patient was asked to be clear whether he or she experienced: a) no unpleasant sensation (score “0”); b) unpleasant sensation (score “1”); c) mild pain (no analgesic used; score “2”) or d) pain (analgesic used; score “3”).

If the patients underwent more than one test, he or she was asked to provide an overall judgement.

### Percutaneous Needle Aspiration Techniques

FNAB. One ml or less of local anaesthetic (lidocaine 2%) was injected subcutaneously. All aspirations were made through a single skin puncture over the nodule using 23, 22, 21 or 20 gauge needles. Cytological smears were air-dried for Giemsa staining or spray-fixed for the Papanicolaou method [8].

LNAB. No incision of skin is performed and the skin is anaesthetized as for FNAB. The syringe can contain heparin to prevent coagulation of blood around the tissue specimen. The needle is inserted into the nodule through the same skin puncture already made for the just performed FNAB. The needle is then rotated within the nodule so that the sharp end severs the tissue fragments, which are then aspirated into the barrel of the syringe. A simpler method to obtain tissue fragments is to perform the same procedure as for FNAB only more vigorously. Needles of different sizes (16–22 gauge, being the 16 gauge needle used only in rare very large nodules) can be used according to the dimensions and consistency of the nodule. LNAB provides tissue fragments of variable size depending on the needle size and nodule pathology. The tissue fragments are usually easily visible [9], [10].

The study was approved by Committee of the Medical Department, Pisa University. Verbal consent was obtained because LNAB is part of our routine work since 1980. A survey documented the process. Verbal informed consent was obtained from all participants. Verbal consent procedure was approved by Ethics Committee of the Medical Department, Pisa University.

## Statistical Analysis

The comparison between treatment groups was performed by non-parametric tests: Fisher’s exact test for qualitative variables and unpaired Wilcoxon test quantitative variables. For the score ordinal variables, the comparison between groups was performed using ordinal logistic regression.

## Results

Table 1 shows the distribution of the sensation score experienced by the patients in the two groups: FNAB or FNAB+LNAB. There were no significant differences between the two groups.

**Table 1:** Table 1 shows the distribution of the sensation score experienced by the patients in the two groups: FNAB or FNAB+LNAB.

SENSATION (score)	FNAB (N [%])	FNAB+LNAB (N [%])
No unpleasant (0)	24 [30.77]	15 [31.25] NS
Unpleasant (1)	31 [39.74]	22 [45.83] NS
Mild pain (2)	22 [28.20]	10 [20.83] NS
Pain (3)	1 [1.29]	1 [2.09] NS
Total	78 [100]	48 [100] NS

There were no significant differences between the two groups.

* NS – nonsignificant differences between the two groups for each sensation score.

Table 2 compares sex distribution, age, number of the nodules, the size of the needles used for FNAB, the number of the repeated test (FNAB or FNAB +LNAB) and the sensation score. In the FNAB group, the distribution of nodule sizes was: up to 1 cm (55%), 1–2 cm (35%), 2–3 cm (7%), and >3 cm (3%). In the FNAB+LNAB group, the distribution of nodule sizes was: up to 1 cm (21%), 1–2 cm (46%), 2–3 cm (19%), and >3 cm (14%). In the FNAB group, the distribution of the needle size used was: 23 gauge (38.5%), 22 gauge (56.4%), and 20 gauge (5.1%). In the FNAB+LNAB group, the distribution of the FNAB needle size was: 22 gauge (34%), 21 gauge (9.5%), and 20 gauge (56.5%) and the distribution of the LNAB needle size was: 22 gauge (2.1%), 18 gauge (54.2%), 20 gauge (37.5%), 21 gauge (4.1%), and 16 gauge (2.1%). Sex distribution, age and the number of the nodules were not significantly different in the 2 groups. As expected, the size of the needle used for FNAB was significantly higher in the FNAB+LNAB group. The proportion of the patients who repeated the test was 37% in the FNA group and 60% in the FNAB+LNAB group (p = 0.016). The number of the tests per patient performed on each group was significantly higher in the FNAB+LNAB group (p = 0.0004). However the pain or discomfort score was not significantly different into the 2 groups using Wilcoxon test (p = 0.587) or the ordinal logistic regression (p = 0.589).

**Table 2:** Principal clinical and technique related parameters in the two patient groups.

PARAMETER	FNAB	FNAB+LNAB	p-value
	N = 78	N = 48	
Sex, women %	87.2	87.5	.958
Age, years, m±SD	59.2±14.6	60.6±15	.531
Nodule, n, m ±SD	1.4±0.6	1.6±0.7	.292
Nodule size, cm, m ±SD	1.4±0.7	2.1±0.9	<0.0001
Needle size, gauge FNAB, m ±SD	22.3±0.7	20.8±1.0	<0.0001
Needle size, gauge LNAB, m ±SD	n/a	18.9±1.2	n/a
Repetition, n, m ±SD	1.6±0.9	2.5±1.4	.0004
Pain score, m ±SD	1±0.8	0.9±1.8	.587

N = number, m = mean, S.D. = standard deviation, n/a = not available.

## Discussion

To our knowledge, there has not been any published data on disomfort or pain following FNAB, LNAB, or LNCB of thyroid nodules.

The results of this study confirm our 32 year experience that LNAB does not produce more discomfort or pain than FNAB. An increase in needle size from 25 to 20 gauge corresponds to an increase of the needle external diameters from 0.5 to 0.9 mm (or 0.4 mm difference) as shown in a previous study [9]. However, in the present study, the mean gauge of the needles used for FNAB in the two groups were 22 (for FNAB) and 21 (for FNA+LNAB), corresponding to a very small difference of the external diameters from 0.19 to 0.24 mm, that is 0.05 mm [9].

The group who underwent FNAB+LNAB received more punctures and the proportion of multiple nodules was slightly higher in the FNA+LNAB group. However the FNAB+LNAB group did not experience more discomfort or pain. This may be due to the degree of subcutaneous anesthesia.

The statement that LNB is associated with increased pain and complications [3] is more applicable to LNCB rather than LNAB. In fact, significant complications, such as large hematomas requiring surgical intervention or laryngeal nerve palsy, have been reported in a few cases following LNCB but not after LNAB [9], [10]. The tolerance and safety of FNAB and LNAB is supported by the present comparison study, a long standing experience on LNAB [8], [9], [11], [12], and microscopic studies on the dimensions of the needles used for FNAB and LNAB [9].

The benefits of incorporating LNAB in the diagnostic algorithm for thyroid nodule evaluation can be summarized as follows.

LNAB has been successfully tested in thousands of patients to reduce surgical interventions for benign thyroid nodules [8]. LNAB has been successfully tested, and compared to FNAB, for reducing the number of inadequate findings following FNAB cytology [11], [12]. LNAB has been successfully tested and compared to FNAB for reducing the number of indeterminate follicular findings following FNAB cytology [8], [11], [13], [14], [15], [16]. LNAB has been successfully tested and compared to FNAB for reducing the number of indeterminate Hürthle cell nodules at FNAB cytology [17]. LNAB has been successfully tested and compared to FNAB for the preoperative diagnostic accuracy of the thyroid nodules [11], [17], [18]. LNAB has been successfully tested and compared to FNAB for providing a more abundant and suitable biological substrate for the determination of thyroid tissue tumor markers [2], [9], [11], [19], [20]. The immunodetection of galectin-3 on LNAB histological specimen from thyroid nodules has shown higher diagnostic accuracy, especially specificity, than FNAB cytology [11], [18], [20].

In conclusion, the present study suggests that concerns of discomfort or pain following LNAB of thyroid nodules may not be generally applicable and therefore in light of the demonstrable benefits, LNAB should be considered as part of the diagnostic algorithm for thyroid nodule evaluation.
